# Molecular Characterization Based on Whole-Genome Sequencing of *Streptococcus pneumoniae* in Children Living in Southwest China During 2017-2019

**DOI:** 10.3389/fcimb.2021.726740

**Published:** 2021-11-02

**Authors:** Ziyi Yan, Yali Cui, Xiaocui Huang, Shikun Lei, Wei Zhou, Wen Tong, Wen Chen, Meijing Shen, Kaifeng Wu, Yongmei Jiang

**Affiliations:** ^1^Department of Laboratory Medicine, West China Second University Hospital, Sichuan University, Chengdu, China; ^2^Key Laboratory of Birth Defects and Related Diseases of Women and Children (Sichuan University), Ministry of Education, Chengdu, China; ^3^Department of Laboratory Medicine, Meishan Women and Children’s Hospital, Alliance Hospital of West China Second University Hospital, Sichuan University, Meishan, China; ^4^Department of Laboratory Medicine, Chengdu Jinjiang District Maternal and Child Healthcare Hospital, Chengdu, China; ^5^State Key Laboratory of Biotherapy, Sichuan University, Chengdu, China; ^6^Department of Laboratory Medicine, Sichuan Jinxin Women and Children Hospital, Chengdu, China; ^7^Department of Pediatrics, West China Second University Hospital, Sichuan University, Chengdu, China; ^8^Department of Laboratory Medicine, Zunyi Medical University Third Affiliated Hospital/First People’s Hospital of Zunyi, Zunyi, China

**Keywords:** *Streptococcus pneumoniae*, whole-genome sequencing, multilocus sequence typing, serotype, antibiotic resistance, China, virulence factors

## Abstract

**Background:**

*Streptococcus pneumoniae* is an important pathogen causing high morbidity and high mortality in children and undergoes frequent recombination for capsule switching to neutralize the 13-valent pneumococcal conjugate vaccine (PCV13). This study aimed to investigate the prevalence, and molecular characteristics including serotypes and antibiotic susceptibility of *S. pneumoniae* isolated from children living in Southwest China from 2017 to 2019 to facilitate the selection of effective vaccine formulations and appropriate antibiotic treatment regimens.

**Methods:**

This study was conducted at West China Second University Hospital (Chengdu, Sichuan Province, China), Zunyi Medical University Third Affiliated Hospital/First People’s Hospital of Zunyi (Zunyi, Guizhou Province, China) and Chengdu Jinjiang District Maternal and Child Healthcare Hospital (Chengdu, Sichuan Province, China). Demographic and clinical characteristics of children infected with *S. pneumoniae* were collected and analysed. Next-generation sequencing and sequence analysis were used to determine the serotypes, sequence types, antibiotic resistance and potential protein vaccine target genes of the pneumococcal isolates. The coverage rate provided by PCV13 was estimated by calculating the percentage of the specific serotypes that were specifically the PCV13-included serotypes. Antimicrobial susceptibility was determined by the microdilution broth method.

**Results:**

The most prevalent pneumococcal serotypes were 19F (25.8%), 19A (14.1%), 6B (12.5%), 6A (9.4%) and 14 (7.8%). The predominant STs were ST271 (23.3%), ST320 (15.5%) and ST90 (8.6%), dominated by the clonal complex Taiwan^19F^-14 (39.1%). The coverage rate of PCV13 was 77.3% in all the isolates, with relatively higher values in invasive isolates (86.4%). Over the decade, the rates of resistance to penicillin, amoxicillin and cefotaxime were 5.6%, 5.3% and 5.1%, respectively, with significantly higher values in invasive isolates (22.4%, 14.9% and 11.9%). Almost all the isolates were resistant to erythromycin (99.1%) and clindamycin (95.9%). All isolates carried virulence-related genes, including *ply, psaA, piaA, piuA, phtE, nanA, pepO, danJ, pvaA, clpP, pcsB, stkP, potD*, and *strH*. The carriage of virulence and resistance genes varied among serotypes and clades, with serotype 19F/ST271 showing higher resistance to antibiotics and being more likely to carry pilus genes and other virulence genes.

**Conclusion:**

These data provide valuable information for the understanding of pneumococcal pathogenesis, antimicrobial resistance and the development of protein-based vaccines against pneumococcal infection.

## Introduction

*Streptococcus pneumoniae* (*S. pneumoniae*, also known as pneumococcus) is a gram-positive, extracellular, opportunistic pathogen that colonizes the mucosal surfaces of the human upper respiratory tract, and up to 27–65% of children and <10% of adults carry this organism (Abdullahi et al., 2012; Yahiaoui et al., 2016). This carriage is the prerequisite for both transmission to other individuals and disease progression in the carrier, including invasive pneumococcal diseases (IPDs, such as meningitis, septicaemia, and pleurisy) and non-invasive pneumococcal diseases (non-IPDs, such as pneumonia, otitis media, and sinusitis) (Weiser et al., 2018). The World Health Organization (WHO) estimated that approximately 800,000 children die of pneumococcal diseases annually, and more than 90% of these deaths occur in developing countries (O'Brien et al., 2009; Johnson et al., 2010).

There are at least 98 serotypes of *S. pneumoniae* circulating worldwide, categorized according to the unique glycan components and linkages that constitute the capsular polysaccharide of each serotype (Yother, 2011), which are the vaccine antigen targets of 13-valent pneumococcal conjugate vaccine (PCV13, including serotypes: 1, 3, 4, 5, 6A, 6B, 7F, 9V, 14, 18C, 19A, 19F and 23F). However, geographical differences in serotype distribution lead to reduced effectiveness of the vaccine when they are implemented in geographic areas where the serotypes are not covered by PCV13 (Balsells et al., 2017). In addition, *S. pneumoniae* is known to undergo frequent recombination (Vos and Didelot, 2009; Mostowy et al., 2017), and capsule switching was reported to occur more frequently following the introduction of pneumococcal vaccines (Chang et al., 2015). Therefore, since PCV13 was introduced in mainland China in 2017, changes in serotype prevalence have been monitored to appropriately choose effective vaccine formulations.

The virulence of *S. pneumoniae* is conferred by capsular polysaccharides and multiple virulence factors, which may vary among clades. Knowledge of the virulence profile of isolates is crucial to predict disease severity and outcome of infection and allows risk assessment during the early onset of the disease (Deurenberg et al., 2017). Moreover, the resistance status is an important reference for antimicrobial selection for anti-infection therapy, and antibiotic resistance varies in different regions, so local antimicrobial resistance surveillance is essential to obtain evidence for clinical practice (Li et al., 2019). Traditionally, studies on virulence and antibiotic resistance usually involve the design of primers for particular genes for PCR assays, which may limit the capacity of molecular characterization analysis. Currently, whole-genome sequencing technology based on next-generation sequencing (NGS) is used to determine the DNA sequence of a complete bacterial genome in a single sequence run, and from these data, information on virulence and antibiotic resistance, as well as information on molecular serotypes and sequence types (STs), is obtained, which is useful for pathogenic surveillance (Deurenberg et al., 2017).

In 2017, the WHO included *S. pneumoniae* as one of 12 priority pathogens, and the continued high burden of pneumococcal disease and rising rates of resistance to antibiotics have renewed interest in disease prevention (Weiser et al., 2018). Moreover, the pandemic of COVID-19 may have impact on the co-infection mode of *S. pneumoniae* and the virus and affect the molecular characteristics of the prevalent strains. Therefore, the baseline levels of the pathogen *S. pneumoniae* before the COVID-19 outbreak will provide important data to support and provide a reference for the investigation of the current status of pneumococcal diseases and possible genetic changes in subsequent prevalent isolates.

In this study, whole-genome NGS technology-based strategies were employed to investigate the prevalence and molecular characteristics of *S. pneumoniae* strains isolated from paediatric patients in Southwest China during 2017-2019. The coverage rates for PCVs were calculated. The virulence protein encoding genes and antibiotic resistance-related genes among *S. pneumoniae* serotypes/STs were analysed and compared.

## Materials and Methods

### Study Area and Population

This study was conducted from March 2017 to November 2019 at West China Second University Hospital (Chengdu, Sichuan Province, China), Zunyi Medical University Third Affiliated Hospital/First People’s Hospital of Zunyi (Zunyi, Guizhou Province, China) and Chengdu Jinjiang District Maternal and Child Healthcare Hospital (Chengdu, Sichuan Province, China). These hospitals are one of China’s largest specialty hospitals for children and women, a general hospital affiliated with a provincial medical university, and a typical community healthcare hospital, respectively. The hospitals’ clinical laboratories have been accredited by the College of American Pathologists (CAP) or the China National Accreditation Service for Conformity Assessment (CNAS) under the ISO15189 accreditation standard or are under the supervision of the above-mentioned external quality assessment laboratory.

The enrolled subjects were children from Southwest China presenting with an *S. pneumoniae* infection who were admitted to these hospitals. The participant eligibility criteria included the following: (1) was younger than 14 years old; (2) gave clinical specimens from which *S. pneumoniae* was isolated and positively cultured; (3) had respiratory, neural, circulatory or local infectious manifestations; and (4) was not vaccinated against *S. pneumoniae*.

To have a comprehensive understanding on the antibiotic susceptibility of *S. pneumoniae*, we expanded the sample size by retrospectively collecting additional antibiotic susceptibility test (AST) results of *S. pneumoniae* isolates from patients (retrospective cohort) that met the participant eligibility criteria between January 2010 and March 2017.

### Isolation and Identification of Strains

The strains of *S. pneumoniae* were collected, isolated and identified in line with the requirements for clinical procedures as previously reported (Yan et al., 2019). In brief, specimens were collected by specialized sample collection personnel or physicians, and the strains were isolated on Columbia agar + 5% sheep blood plates (BD Medical Technology, NJ, USA), which were incubated at 35°C for 24–48 h in a 5% carbon dioxide (CO_2_) environment. All isolates were identified by typical colony morphology and optochin assays, and the results were confirmed by matrix-assisted laser desorption ionization time-of-flight mass spectrometry (MALDI-TOF MS; Vitek MS system; BioMerieux, Rhône, France). Strains isolated from sterile sites e.g. cerebrospinal fluid, blood and pleural fluid, were defined as IPD strains. Pneumococcal isolates were stored in 25% sterile glycerol broth at −70°C for subsequent analysis.

### Genome Sequencing, Assembly, and Annotation

Genomic DNA was extracted by the QIAamp DNA Minikit (Qiagen, Hilden, Germany), and sequencing libraries were generated using the NEBNext^®^ Ultra™ DNA Library Prep Kit for Illumina (New England Biolabs, NEB, USA) following the manufacturer’s recommendations. Then, the whole genomes of *S. pneumoniae* were sequenced using the Illumina NovaSeq PE150 platform (Illumina, San Diego, CA, USA) with approximately 200× coverage at Beijing Novogene Bioinformatics Technology Co., Ltd. The genome data were assembled with SPAdes software (v3.14.1) (Prjibelski et al., 2020), and different K-mers (21, 33, 55, 77) were selected for assembly to obtain the assembly result with the optimal k-mer value and the fewest scaffolds. All of the assembled genomes were submitted to GenBank and approved (PRJNA656156, PRJNA681770 and PRJNA643306). The genome of *S. pneumoniae* R6 (GenBank: AE007317.1) was used to annotate the assembled genome with Prokka software (v1.14.5) (Seemann, 2014).

### Molecular Serotyping

Molecular serotypes were identified by pneumococcal capsule typing (PneumoCaT v1.2.1; https://github.com/phe-bioinformatics/PneumoCaT), written in Python (v2.7.6), utilizing a two-step method to assign capsular type (Kapatai et al., 2016). In brief, reads from each readset were mapped to capsular locus sequences for all known capsular types. If more than 1 locus was matched, then a variant-based approach utilized the capsular type variant (CTV) database to distinguish serotypes within a serogroup/genogroup.

### Multilocus Sequence Typing

Multilocus sequence typing (MLST) was performed to determine the STs of the isolates. Seven housekeeping genes (*aroE, gdh, gki, recP, spi, xpt, ddl*) were compared with the pneumococcal MLST database (https://pubmlst.org/organisms/streptococcus-pneumoniae) through mlst software (v2.19.0) (Seemann T, mlst, GitHub, https://github.com/tseemann/mlst) (Jolley and Maiden, 2010). Novel alleles were amplified by PCR and sequenced by the Sanger method (ABI3730XL, Sangon Biotech, Shanghai, China) for verification (Yan et al., 2019), and the sequences were submitted to the pneumococcal MLST database to assign new numbers. The minimum spanning tree-like structures were illustrated by PHYLOVIZ software (version 2.1, http://www.phyloviz.net) *via* goeBURST Full MST (goeBURST distance) at level 1 (SLVs) and level 6 (Feil et al., 2004; Francisco et al., 2012).

### Phylogenetic Analysis

The general feature format (gff) files of 128 isolates produced by Prokka were analysed using Roary v3.13.0 to create a multiFASTA alignment of core genes (>99%) using MAFFT (Page et al., 2015). Snp-sites (v2.3.3, https://github.com/andrewjpage/snp-sites) was used to delete duplicate sites in the multiFASTA alignment. A maximum-likelihood tree was constructed from the alignment produced by RAxML (v8.2.10, https://github.com/stamatak/standard-RAxML) using the GTRGAMMA method (Stamatakis, 2014). Finally, the RAxML tree was visualized and annotated in iTOL (v6, https://itol.embl.de).

### Gene Analysis

Virulence genes, antimicrobial resistance genes and recombinant protein vaccine target-related genes were screened by ABRicate software (v1.0.1) (Seemann T, Abricate, GitHub https://github.com/tseemann/abricate) *via* VFDB, NCBI AMRFinderPlus and an in-house sequence database, respectively (Chen et al., 2016; Feldgarden et al., 2019; Yan, 2021). According to previous reports (Hakenbeck et al., 2012; Zhou et al., 2016), prominent amino acid substitutions in penicillin (PEN)-binding proteins (PBP1a, PBP2b and PBP2x) were analysed by MEGA (v 7.0, http://www.megasoftware.net) in strains with MICs <0.06 µg/mL or in those resistant to β-lactams.

### Antibiotic Susceptibility Tests

ASTs were performed based on turbidimetry using AST cards (BioMerieux, Rhône, France). The antimicrobial agents included PEN, amoxicillin (AMX), ceftriaxone (CRO), cefotaxime (CTX), cefuroxime (CXM), meropenem (MEM), levofloxacin (LVX), moxifloxacin (MXF), erythromycin (ERY), quinupristin/dalfopristin (Synercid, QD), clindamycin (CLI), tetracycline (TET), chloramphenicol (CHL), vancomycin (VAN) and trimethoprim-sulfamethoxazole (SXT). Quality control analysis was performed using *S. pneumoniae* ATCC49619. The operational processes and interpretation of the results were performed according to the manufacturer’s instructions and the Clinical and Laboratory Standards Institute (CLSI) 2021 standard (Weinstein, 2021).

### Statistical Analysis

Statistical Package for Social Science (SPSS) software for Windows was used to assess the statistical significance of the data (version 22.0; Chicago, IL, USA). The chi-square test, Fisher’s exact test and T-test were used. On the basis of the chi-square test, the Bonferroni method was used to test whether the differences among multiple groups were statistically significant, and P values < 0.05 were considered statistically significant.

### Ethics Statement

The clinical experimental plan was approved by the Clinical Trial Ethics Committee of West China Second University Hospital, Sichuan University (No. 2018021). Before enrolment, written informed consent was obtained from legal guardians on behalf of the children involved in the study. The work was carried out in accordance with the Declaration of Helsinki.

## Results

### Demographic and Clinical Characteristics

A total of 128 patients (prospective cohort) from 26 cities were enrolled from March 2017 to November 2019. Fifty-nine IPD cases and 69 non-IPD cases were included, with ages ranging from 0 to 14 years and a median age (P25-P75) of 1.17 (0.75-3.00) years. The pneumococcal diseases included meningitis, bacteraemia, pleural and peritoneal inflammation, pneumonia, bronchopneumonia, upper respiratory tract infections and otitis media. In total, 121 patients (94.5%) had a favourable prognosis.

To have a comprehensive understanding on the antibiotic resistance phenotype, we retrospectively collected additional AST results of pneumococcal isolates from 993 cases (retrospective cohort) admitted at hospitals between January 2010 and March 2017. Overall, 926 non-IPD cases and 67 IPD cases were included, with a median age (P25-P75) of 1.00 (0.58-2.00) years (**Table 1**). The demographic and clinical characteristics of the patients were summarized in **Table 1**. No significant differences in those characteristics were observed between the two cohorts.

**Table 1:** Demographic and clinical characteristics.

Characteristics	Prospective		Retrospective	
	No. of patients	%	No. of AST cases	%
*Total*	128	100.0	993	100.0
*Gender*				
Male	77	60.2	654	65.9
Female	51	39.8	339	34.1
*Age (years)*				
* Median age (P25-P75)*	1.17 (0.75-3.00)		1.00 (0.58-2.00)	
<1	41	32.0	428	43.1
1-2	49	38.2	373	37.6
3-5	24	18.9	143	14.4
6-14	14	10.9	49	4.9
*Primary discharge diagnosis*				
Meningitis	12	9.4	20	2.0
Bacteraemia	41	32.0	42	4.2
Pleural inflammation	5	3.9	5	0.5
Peritoneal inflammation	1	0.8	0	0.0
Severe pneumonia	10	7.8	33	3.3
Pneumonia (ordinary)	32	25.0	446	44.9
Bronchitis	5	3.9	46	4.6
Bronchopneumonia	13	10.2	334	33.6
Upper respiratory infections	3	2.3	17	1.7
Otitis media	6	4.6	19	1.9
Others	0	0.0	34	3.4
*Sample type*				
Cerebrospinal fluid	12	9.4	20	2.0
Blood	40	31.2	42	4.2
Pleural fluid	5	3.9	5	0.5
Ascites	2	1.6	0	0.0
Alveolar lavage fluid	8	6.2	18	1.8
Sputum	54	42.3	889	89.2
Endotracheal tube tip	1	0.8	0	0.0
Secretions	6	4.6	19	1.9
* invasive pneumococcal disease*	59	46.1	67	6.7
* non-invasive pneumococcal disease*	69	53.9	926	93.2
*Prognosis*				
Cured	91	71.1		
Improved and transferred	30	23.4		
Exacerbation and transferred	2	1.6		
Sequela	2	1.6		
Dead/Abandoned	3	2.3		

### Molecular Serotyping and Vaccine Coverage Rates

**Figure 1:**
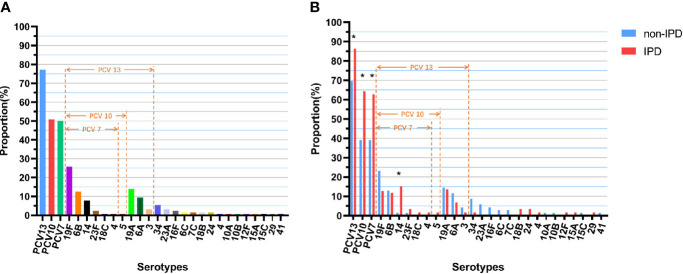
Serotype distribution and coverage of PCVs among *S. pneumoniae* isolates. **(A)** Proportions of each serotype in all 128 isolates. **(B)** Proportions of each serotype in different diseases (non-IPD: n = 69; IPD: n = 59). “*” indicates a significant difference between the proportions (p < 0.05).

All 128 isolates were successfully serotyped, and 24 different serotypes were identified. The most prevalent serotypes were 19F (25.8%), 19A (14.1%), 6B (12.5%), 6A (9.4%), 14 (7.8%) and 34 (5.5%). Of the 128 isolates, 64 (50.0%) were the PCV7 vaccine serotypes, 65 (50.8%) were the PCV10 vaccine serotypes, and 99 (70.3%) were the PCV13 vaccine serotypes (**Figure 1A**). The most prevalent *S. pneumoniae* IPD serotypes were 19F (28.8%), 14 (15.3%), 19A (13.6%), 6B (11.9%), 6A (6.8%) and 23F (3.4%); the coverage rates forPCV7, PCV10 and PCV13 were 50.0%, 50.8% and 77.3%, respectively, which were significantly higher than those for non-IPD strains (PCV7: 62.7% vs. 39.1%, p=0.008; PCV10: 64.4% vs. 39.1%, p=0.004; PCV13: 86.4% vs. 69.6%, p=0.023) (**Figure 1B**). In addition, serotype 14 was more often isolated in patients with IPD than non-IPD patients (15.3% vs. 1.4%, p=0.004).

### Multilocus Sequence Typing

**Figure 2:**
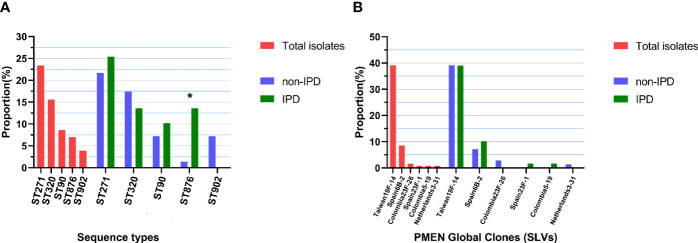
Top 5 ST distributions and PMEN clone proportions. **(A)** Proportions of each of the top 5 STs in all 128 isolates and in different diseases (non-IPD: n = 69; IPD: n = 59), “*” indicates a significant difference between the composition ratios (p < 0.05). **(B)** Proportions of each PMEN global clone in all 128 isolates and in different diseases (non-IPD: n = 69; IPD: n = 59).

Forty-seven different STs were identified by MLST analysis in 128 isolates, including 8 novel STs (ST16211, ST16327, ST16328, ST16329, ST16330, ST16423, ST16424, ST16425) and 3 novel alleles (ddl [1059], gdh [711], gdh [712]). The predominant ST was ST271 (n=30, 23.3%), followed by ST320 (n=20, 15.5%), ST90 (n=11, 8.6%), ST876 (n=9, 7.0%) and ST902 (n=5, 3.9%). Other STs collectively accounted for 48.7% (**Figure 2A**).

**Figure 3:**
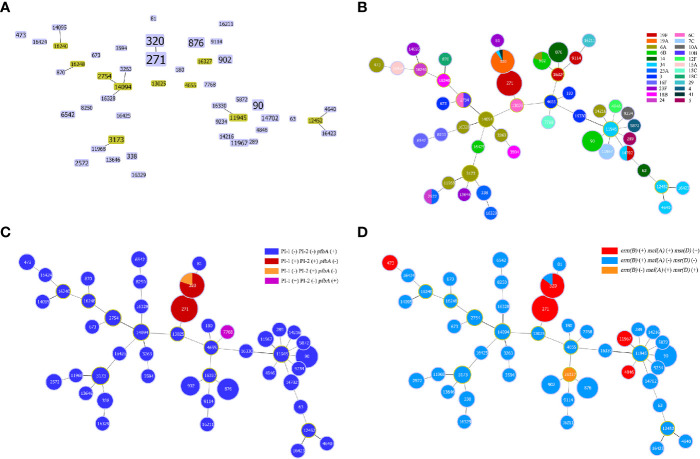
Minimum spanning tree-like structure *via* the goeBURST full MST algorithm. **(A)** goeBURST distance of level 1 (SLVs) showing that the 47 STs were divided into 7 CCs (linked) and 28 singletons. **(B)** goeBURST distance of level 6 showing the relationship between STs and serotypes. Each disk represents an ST, and each colour represents a serotype. **(C)** The relationship between STs and pilus/pfbA, and that between pilus and pfbA, was mutually exclusive, while PI-I and PI-II were closely linked. **(D)** Relationship between STs and ERY resistance-related genes.

By comparing the strains with the pneumococcal molecular epidemiology network (PMEN) clones, six global clones (with at least 6 of 7 MLST alleles shared) were found in this study, including Taiwan^19F^-14 (n=50, 39.1%), Spain^6B^-2 (n=11, 8.6%), Colombia^23F^-26 (n=2, 1.6%), Colombia^5^-19 (n=1, 0.8%), Netherlands^3^-31 (n=1, 0.8%) and Spain^23F^-1 (n=1, 0.8%). The strains belonging to these global clones or their single-locus variants (SLVs) made up 51.6% (n=66) of all strains (**Figure 2B**). Seven clonal complexes (CCs) and 28 singletons were obtained *via* goeBURST distance analysis (SLV, level 1, **Figure 3A**). CC271 (including ST271 and ST320) was the most prevalent CC which accounted for 39.1% (n=50/128) of the strains.

Strains of the same serotype showed an aggregation trend in MLST analysis *via* the goeBURST Full MST algorithm, and the minimum spanning tree-like structure indicated that ST271, ST320, ST90, ST902 and ST876 were associated with serotypes 19F, 19A, 6B, 6B and 14, respectively (**Figure 3B**). In addition, ST876 was more frequently found among IPD isolates than non-IPD isolates (13.6% *vs.* 1.4%, p=0.012, **Figure 2A**).

### Prevalence of Virulence Genes

All 128 isolates carried *pce* (*cbpE*), *pavA*, *lmb*, *srtA*, *slrA*, *plr (gapA)*, *nanA*, *eno*, *piaA*, *piuA*, *psaA*, *cppA*, *htrA (degP)*, *tig (ropA)*, and *ply*. Most of the isolates carried *lytA* (99.2%), *lytC* (99.2%), *hysA* (98.4%), *cbpD* (93.0%), *lytB* (92.2%) and *nanB* (85.2%). The presence of these genes suggests that all clinical isolates have adherence-, exoenzyme-, iron uptake-, manganese uptake-, protease- and toxin-related genes. In addition, more than half of the strains carried *iga* (64.1%) and *pfbA* (60.9%), and less than half of the strains carried PI-I (36.7%), PI-II (39.1%), *cbpG* (35.9%), *zmpB* (14.1%) and *zmpC* (3.9%). The detailed information can be found in **Table 2**.

**Table 2:** Characteristics of virulence genesin 128 isolates.

Virulence genes	Total	%	Disease^a^					Serotypes													Clonal complexes									
			Non-IPD	%	IPD	%	P value	19F	%	19A	%	6B	%	6A	%	14	%	Others	%	P value	CC271		%	CC90	%	CC876	%	Others	%	P value
**Adherence**																														
*cbpD*	119	93.0	62	89.9	57	96.6	0.136	32	97.0	16	88.9	16	100.0	9	75.0	10	100.0	36	92.3	0.102	47		94.0	11	100.0	9	100.0	52	89.7	0.463
*cbpG*	46	35.9	30	43.5	16	27.1	0.055	**2†**	**6.1†**	**0†**	**0.0†**	**15§**	**93.8§**	**4¶**	**33.3**	**1**	**10.0**	**24**	**61.5**	0.000*	**0†**		**0.0†**	**11§**	**100.0§**	**0†**	**0.0†**	**35§**	**60.3§**	0.000*
*pce*	128	100.0	69	100.0	59	100.0		33	100.0	18	100.0	16	100.0	12	100.0	10	100.0	39	100.0		50		100.0	11	100.0	9	100.0	58	100.0	
*lytA*	127	99.2	68	98.6	59	100.0	0.353	33	100.0	18	100.0	16	100.0	11	91.7	10	100.0	39	100.0	0.083	50		100.0	11	100.0	9	100.0	57	98.3	0.749
*lytB*	118	92.2	65	94.2	53	89.8	0.358	30	90.9	18	100.0	16	100.0	11	91.7	10	100.0	33	84.6	0.227	**50§**		**100.0§**	**11§**	**100.0§**	**9§**	**100.0§**	**48†**	**82.8†**	0.004*
*lytC*	127	99.2	68	98.6	59	100.0	0.353	33	100.0	18	100.0	16	100.0	12	100.0	10	100.0	38	97.4		50		100.0	11	100.0	9	100.0	57	98.3	0.749
*pavA*	128	100.0	69	100.0	59	100.0		33	100.0	18	100.0	16	100.0	12	100.0	10	100.0	39	100.0		50		100.0	11	100.0	9	100.0	58	100.0	
*pfbA*	78	69.0	42	60.9	36	61.0	0.986	**3†**	**9.1†**	**0†**	**0.0†**	**16§**	**100.0§**	**12§**	**100.0§**	**10^§^**	**100.0§**	**37§**	**94.9§**	0.000*	**0†**		**0.0†**	**11§**	**100.0§**	**9§**	**100.0§**	**58§**	**100.0§**	0.000*
*lmb*	128	100.0	69	100.0	59	100.0		33	100.0	18	100.0	16	100.0	12	100.0	10	100.0	39	100.0		50		100.0	11	100.0	9	100.0	58	100.0	
*srtA*	128	100.0	69	100.0	59	100.0		33	100.0	18	100.0	16	100.0	12	100.0	10	100.0	39	100.0		50		100.0	11	100.0	9	100.0	58	100.0	
*slrA*	128	100.0	69	100.0	59	100.0		33	100.0	18	100.0	16	100.0	12	100.0	10	100.0	39	100.0		50		100.0	11	100.0	9	100.0	58	100.0	
*plr/gapA*	128	100.0	69	100.0	59	100.0		33	100.0	18	100.0	16	100.0	12	100.0	10	100.0	39	100.0		50		100.0	11	100.0	9	100.0	58	100.0	
**Pilus**																														
PI-1 ^b^	48	37.5	26	37.7	22	37.3	0.963	**30§**	**90.9§**	**15§**	**83.3§**	**0†**	**0.0†**	**0†**	**0.0†**	**0†**	**0.0†**	**3†**	**7.7†**	0.000*	**47§**		**94.0§**	**0†**	**0.0†**	**0†**	**0.0†**	**1†**	**1.7†**	0.000*
PI-2 ^c^	50	39.1	27	39.1	23	39.0	0.986	**30§**	**90.9§**	**18§**	**100.0§**	**0†**	**0.0†**	**0†**	**0.0†**	**0†**	**0.0†**	**2†**	**5.1†**	0.000*	**50§**		**100.0§**	**0†**	**0.0†**	**0†**	**0.0†**	**0†**	**0.0†**	0.000*
**Enzyme**																														
*hysA*	126	98.4	68	98.6	58	98.3	0.911	33	100.0	18	100.0	16	100.0	11	91.7	10	100.0	38	97.4	0.409	50		100.0	11	10.0	9	100.0	56	96.6	0.643
*nanA*	128	100.0	69	100.0	59	100.0		33	100.0	18	100.0	16	100.0	12	100.0	10	100.0	39	100.0		50		100.0	11	100.0	9	100.0	58	100.0	
*nanB*	109	85.2	61	88.4	48	81.4	0.263	**33§**	**100.0§**	**18§**	**100.0§**	**5†**	**31.3†**	**10**	**83.3**	**10^§^**	**100.0§**	**33§**	**84.6§**	0.000*	**50§**		**100.0§**	**0†**	**0.0†**	**9§**	**100.0§**	**50**	**86.2**	0.000*
*eno*	128	100.0	69	100.0	59	100.0		33	100.0	18	100.0	16	100.0	12	100.0	10	100.0	39	100.0		50		100.0	11	100.0	9	100.0	58	100.0	
**Iron uptake**																														
*piaA*	128	100.0	69	100.0	59	100.0		33	100.0	18	100.0	16	100.0	12	100.0	10	100.0	39	100.0		50		100.0	11	100.0	9	100.0	58	100.0	
*piuA*	128	100.0	69	100.0	59	100.0		33	100.0	18	100.0	16	100.0	12	100.0	10	100.0	39	100.0		50		100.0	11	100.0	9	100.0	58	100.0	
**Manganese uptake**																														
*psaA*	128	100.0	69	100.0	59	100.0		33	100.0	18	100.0	16	100.0	12	100.0	10	100.0	39	100.0		50		100.0	11	100.0	9	100.0	58	100.0	
**Protease**																														
*cppA*	128	100.0	69	100.0	59	100.0		33	100.0	18	100.0	16	100.0	12	100.0	10	100.0	39	100.0		50		100.0	11	100.0	9	100.0	58	100.0	
*iga*	82	64.1	44	63.8	38	64.4	0.940	**33§**	**100.0§**	**16**	**88.9**	**11**	**68.8**	**6**	**50.0**	**0†**	**0†**	**16**	**61.0**	0.000*	**48§**		**96.0§**	**11§**	**100.0§**	**0†**	**0†**	**23†**	**39.7†**	0.000*
*htrA/degP*	128	100.0	69	100.0	59	100.0		33	100.0	18	100.0	16	100.0	12	100.0	10	100.0	39	100.0		50		100.0	11	100.0	9	100.0	58	100.0	
*tig/ropA*	128	100.0						33	100.0	18	100.0	16	100.0	12	100.0	10	100.0	39	100.0		50		100.0	11	100.0	9	100.0	58	100.0	
*zmpB*	18	14.1	12	17.4	6	10.2	0.241	**0†**	**0†**	**0†**	**0†**	**1**	**6.3**	**4§**	**33.3§**	**0†**	**0†**	**13§**	**33.3§**	0.000*	**0†**		**0.0†**	**0†**	**0.0†**	**0†**	**0.0†**	**18§**	**31.0§**	0.000*
*zmpC*	5	3.9	3	4.3	2	3.4	0.780	0	0.0	0	0.0	0	0.0	0	0.0	1	10.0	4	10.3	0.135	0		0.0	0	0.0	0	0.0	5	8.6	0.130
**Toxin**																														
*ply*	128	100.0	69	100.0	59	100.0		33	100.0	18	100.0	16	100.0	12	100.0	10	100.0	39	100.0		50		100.0	11	100.0	9	100.0	58	100.0	
**Total**	128		69		59			33		18		16		12		10		39			50			11		9		58		

^a^The classification of disease types combines clinical manifestations and pathogenic diagnosis and ultimately depends on the source of the strain. All patients identified as IPD had S. pneumoniae isolated from their normally sterile sites (cerebrospinal fluid/blood/pleural fluid/ascites). ^b^When rrgA, rrgB, rrgC, srtC-1, srtC-2 and srtC-3 were all detected, the isolate was judged as pilus-Ⅰ (PI-1) positive. When srtA, pitB, sipA, srtG1, and srtG2 were all detected, the isolate was judged as pilus-Ⅱ (PI-2) positive. % Indicates the proportion of the gene detected in the subgroup. Bold indicates that the difference is statistically significant. On the basis of the chi-square test, the Bonferroni method was used to test whether the differences among multiple groups were statistically significant, and the symbols †, § and are used to indicate groups with significant differences (§, high; ¶, medium; †, low). * indicates P value < 0.05.

**Figure 4:**
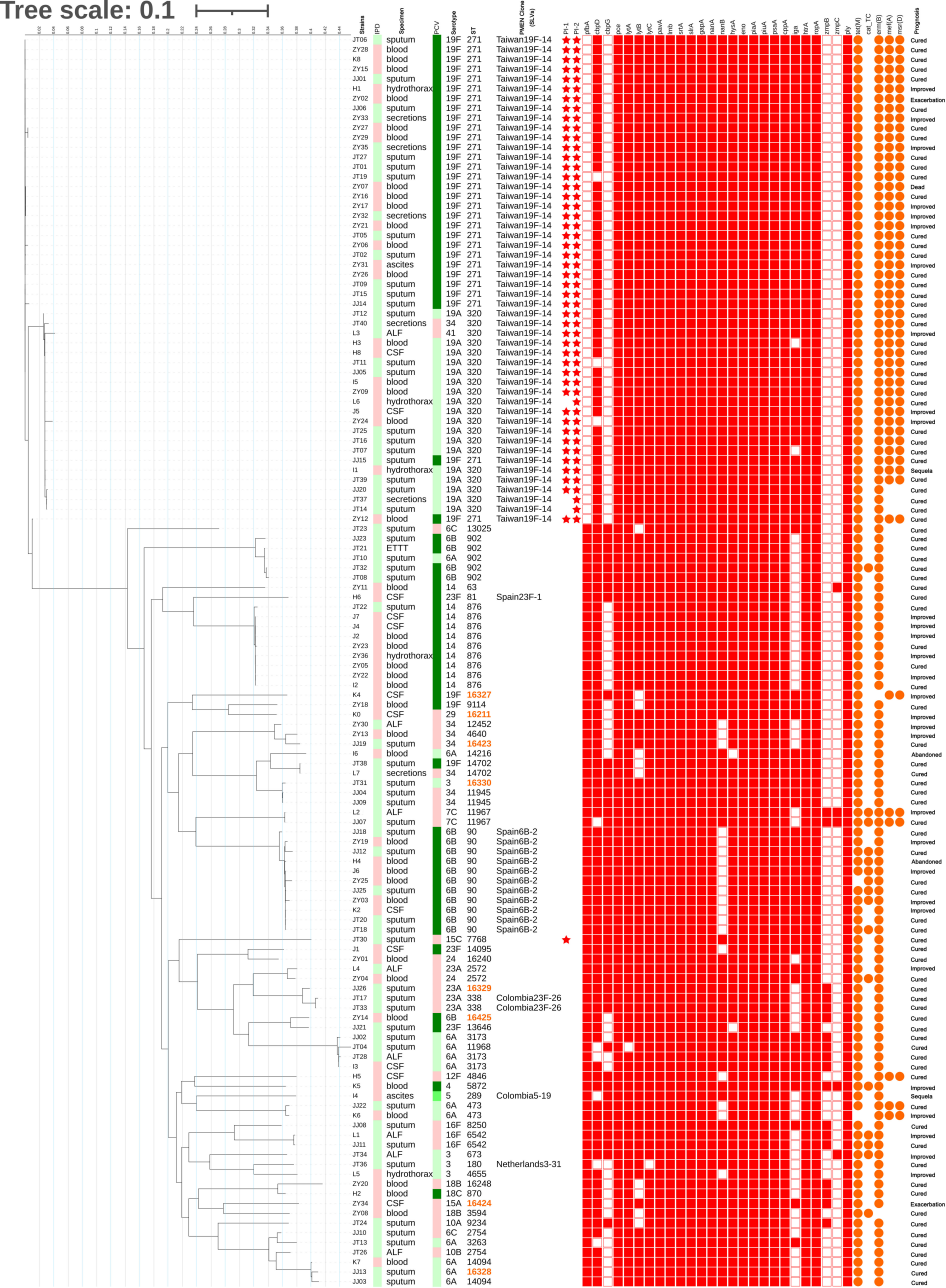
Maximum likelihood tree of 128 isolates. The RAxML tree was constructed from a multiFASTA alignment of core genes (>99%) using the GTRGAMMA method. The genetic characteristics of all isolates were annotated. IPD: Red indicates IPD; green indicates non-IPD. PCV: The green gradient indicates the serotype under the coverage of PCV7, PCV10 and PCV13(from lighter to darker); red indicates the serotype outside the coverage of PCV. ST: Orange indicates the novel STs found in this study. PMEN Clone: CCs related to PMEN global clones at the SLV level. Star: Red star indicates carriage of the pilus. Square: Red filled square indicates carriage of the virulence gene. Circle: Orange filled circle indicates carriage of the resistance-related gene.

The relationship between the isolates and virulence genes is shown in the annotation of the RAxML tree (**Figure 4**). Gene carriage rate varied significantly among strains of different serotypes. For adherence factors, the carriage rate of *cbpG* in serotype 6B was significantly higher than that of other serotypes (93.8% *vs.* 27.7%, p=0.000), and the carriage rate of PI-1 was highest in serotype 19A/19F (88.2% *vs.* 3.9%, p=0.000), which was similar to that of PI-2 (94.1% *vs.* 2.6%, p=0.000). *pfbA* was rarely detected in serotype 19A/19F but was prevalent in other serotypes (5.9% *vs.* 97.4%, p=0.000). For exoenzymes, the carriage rate of *nanB* was lowest in serotype 6B but highest in serotype 19A/19F/14 (31.3% *vs.* 84.3% *vs.* 100.0%, p=0.000). For protease, the carriage rate of *iga* was highest in serotype 19F but lowest in serotype 14 (100.0% *vs.* 57.6% *vs.* 0.0%, p=0.000).

The carriage rate of virulence genes also varied according to CC. For adherence factors, the carriage rate of *cbpG* was highest in CC90 but lowest in CC271/CC876 (100.0% vs. 60.3% *vs.* 0.0%, p=0.000), and *lytB* in CC271/CC90/CC876 was significantly higher than that in other serotypes (100.0% *vs.* 82.8%, p=0.004). The carriage rate of PI-1 was highest in CC271 (94.0% *vs.* 1.3%, p=0.000), which was similar to that of PI-2 (100.0% *vs.* 0.0%, p=0.000). However, *pfbA* was rarely detected in CC271 but was prevalent in other CCs (0.0% *vs.* 100.0%, p=0.000). For exoenzymes, the carriage rate of *nanB* was lowest in serotype CC90 but highest in CC271/CC876 (0.0% vs. 86.2% *vs.* 100.0%, p=0.000). For protease, the carriage rate of *iga* was highest in CC271/CC90 but lowest in CC876 (96.7% *vs.* 39.7% *vs.* 0.0%, p=0.000). It was found that pilus and *pfbA* were mutually exclusive, while PI-I and PI-II were closely linked (**Figure 3C**). All strains in this study carried either PI-II or *pfbA* but not both. In addition, the presence of virulence genes did not show a significant difference among the isolates obtained from IPD and non-IPD patients in this study.

### Conservation of Protein Vaccine Candidate Genes

Twenty-seven protein vaccine candidate genes were found in more than 98% strains of the 128 isolates in this study. Except for a few isolates that did not carry *lytA*, *lytC*, *hysA* and *strH* (carriage: 99.2%, 99.2%, 98.4% and 99.2%, respectively), all isolates carried protein vaccine candidate encoding genes, including *pce*, *ply*, *psaA*, *piaA*, *piuA*, *phtE*, *nanA*, *hysA*, *tuf*, *pepO*, *danJ*, *papP*, *prtA*, *pvaA*, *gndA*, *clpP*, *ppmA*, *eng*, *pcsB*, *stkP*, *potD*, *pgdA*, *strH*, *bgaA* and *pppA*, the majority of which were protective either alone or in combination against pneumococcal infections. Moreover, the identity of the sequences was more than 98% for the candidate *lytC*, *ply*, *psaA*, *piaA*, *piuA*, *phtE*, *tuf*, *pepO*, *danJ*, *gndA*, *clpP*, *ppmA*, *pcsB*, *stkP*, *potD* and *pgdA* genes. The detailed data of recombinant protein vaccine target gene analysis are shown in **Table 3**.

**Table 3:** Characteristics of recombinant protein vaccine target-related genes in 128 isolates.

Protein vaccine target genes	Carriage (%)	Identity (%)	Average identity (%)	Total base pairs	Reference sequence	Literature
*pce*	100.0	>94.0	97.5	1884bp	SP_0930 (TIGR4)	(Gosink et al., 2000)
*lytA*	99.2	>86.3	98.7	957bp	SP_1937 (TIGR4)	(Gosink et al., 2000)
*lytC*	99.2	>99.9	100.0	1473bp	SP_1573 (TIGR4)	(Gosink et al., 2000)
*ply*	100.0	>98.8	99.7	1416bp	SP_1923 (TIGR4)	(Paton et al., 1983)
*psaA*	100.0	>99.4	99.6	930bp	SP_1650 (TIGR4)	(Talkington et al., 1996)
*piaA*	100.0	>98.8	99.9	1026bp	SPD0915 (D39)	(Brown et al., 2001)
*piuA*	100.0	>99.2	99.6	966bp	SPD1652 (D39)	(Brown et al., 2001)
*phtE*	100.0	>99.1	99.4	3120bp	SP_1004 (TIGR4)	(Adamou et al., 2001)
*nanA*	100.0	>88.3	95.1	2222bp	SP_1326 (TIGR4)	(Tong et al., 2005)
*hysA*	98.4	>95.2	99.4	3201bp	SP_0314 (TIGR4)	(Anderson et al., 2016)
*tuf*	100.0	>99.8	99.9	1197bp	SPR1343 (R6)	(Nagai et al., 2019)
*pepO*	100.0	>99.5	99.7	1893bp	SPR1491 (R6)	(Zhang et al., 2016)
*danJ*	100.0	>98.6	99.3	1137bp	SP_0519 (TIGR4)	(Su et al., 2017)
*papP*	100.0	>93.2	99.1	936bp	SP_1298 (TIGR4)	(Kuipers et al., 2016)
*prtA*	100.0	>94.3	96.5	6423bp	SP_0641 (TIGR4)	(Wizemann et al., 2001)
*pvaA*	100.0	>92.0	99.5	615bp	SPR0930 (R6)	(González et al., 2017)
*gndA*	100.0	>98.3	98.6	1425bp	SP_0375 (TIGR4)	(Daniely et al., 2006)
*clpP*	100.0	>99.0	99.5	591bp	SPR0656 (R6)	(Kwon et al., 2004)
*ppmA*	100.0	>99.6	99.8	942bp	SPR0884 (R6)	(Overweg et al., 2000)
*eng*	100.0	>96.3	97.9	5304bp	SP_0368 (TIGR4)	(Anderson et al., 2016)
*pcsB*	100.0	>99.8	99.9	1179bp	SP_2216 (TIGR4)	(Giefing et al., 2008)
*stkP*	100.0	>98.5	99.1	1980bp	SP_1732 (TIGR4)	(Giefing et al., 2008)
*potD*	100.0	>99.4	99.7	1071bp	SP_1386 (TIGR4)	(Ware et al., 2006)
*pgdA*	100.0	>98.7	99.5	1392bp	SP_1479 (TIGR4)	(Vollmer et al., 2002)
*strH*	99.2	>97.2	99.3	3939bp	SP_0057 (TIGR4)	(Dalia et al., 2010)
*bgaA*	100.0	>96.6	98.2	6702bp	SP_0648 (TIGR4)	(Dalia et al., 2010)
*pppA*	100.0	>86.0	95.8	537bp	SP_1572 (TIGR4)	(Green et al., 2005)

### The Relationship Between Antibiotic Susceptibility and Molecular Characteristics of the 128 Isolates

The overall prevalence of PEN-non-susceptible *S. pneumoniae* (PNSP) was 9.4%, including PEN-intermediate *S. pneumoniae* (PISP, 3.9%) and PEN-resistant *S. pneumoniae* (PRSP, 5.5%) during 2017-2019. Among them, the PEN resistance rate of the strains isolated from meningitis patients was significantly higher than that of strains isolated from non-meningitis patients (50.0% *vs.* 0.9%, p=0.000). The detailed data are shown in **Table 4**. Most isolates showed high resistance to ERY (96.9%) and CLI (94.5%), and a considerable number of isolates were resistant to TET (78.9%) and SXT (62.5%). Few isolates were resistant to chloramphenicol (CHL, 11.7%), CRO (11.7%), CTX (10.9%), AMX (4.7%), LVX (3.1%) and MXF (0.8%). No isolate was found to have MEM and VAN resistant phenotypes. Approximately 89.1% (114/128) of the isolates were defined as multidrug resistant (MDR). In addition, isolates from patients with IPD had a higher rate of resistance to PEN (10.2% *vs.* 1.4%, p=0.042) and AMX (10.2% *vs.* 0%, p=0.014). Among the PSSP isolates, isolates resistant to CRO and CTX were observed.

**Table 4:** Antibiotic resistance of *S. pneumoniae* isolates from 2017 to 2019 (prospective study part, n=128).

Resistance phenotypes/genes	Total^b^	%^c^	Diseases^a^					Penicillin resistance					Serotypes			STs		
			Non-IPD	%	IPD	%	P value	PSSP	%	PNSP	%	P value	19F	%	P value^e^	ST271	%	P value^e^
PSSP	116	90.6	64	92.8	52	88.1	0.372						**27**	**81.8**	**0.028**	25	83.3	0.150
PISP	5	3.9	4	5.8	1	1.7	0.373						**4**	**12.1**	**0.044**	**4**	**13.3**	**0.011**
PRSP	7	5.5	**1**	**1.4**	**6**	**10.2**	**0.048**						**2**	**6.1**	**0.016**	1	3.3	0.687
AMX	6	4.7	**0**	**0.0**	**6**	**10.2**	**0.014**	**0**	**0.0**	**6**	**50.0**	**0.000**	1	3.0	1.000	0	0.0	0.335
CRO	15	11.7	8	11.6	7	11.9	0.962	**11**	**9.5**	**4**	**33.3**	**0.000**	**11**	**33.3**	**0.000**	**10**	**33.3**	**0.000**
CTX	14	10.9	8	11.6	6	10.2	0.797	**8**	**6.9**	**6**	**50.0**	**0.000**	**12**	**36.4**	**0.000**	**11**	**36.7**	**0.000**
MEM	0	0.0	0	0.0	0	0.0		0	0.0	0	0.0		0	0.0		0	0.0	
LVX	4	3.1	3	4.3	1	1.7	0.624	4	3.4	0	0.0	1.000	3	9.1	0.052	**3**	**10.0**	**0.040**
MXF	1	0.8	1	1.4	0	0.0	1.000	1	0.9	0	0.00	1.000	0	0.0	1.000	0	0.0	1.000
SXT	80	62.5	46	66.7	34	57.6	0.292	72	62.1	8	66.7	0.754	**27**	**81.8**	**0.011**	**24**	**80.0**	**0.031**
CLI	121	94.5	67	97.1	54	91.5	0.167	110	94.8	11	91.7	0.647	30	90.9	0.373	28	93.3	0.666
VAN	0	0.0	0	0.0	0	0.0		0	0.0	0	0.0		0	0.0		0	0.0	
TET	101	78.9	**60**	**87.0**	**41**	**69.5**	**0.018**	94	81.0	7	58.3	0.128	29	87.9	0.215	26	86.70%	0.310
*tet (M)*	126	98.4	69	100	57	96.6	0.211	114	98.3	12	100	1.000	33	100	1.000	30	100	1.000
CHL	15	11.7	8	11.6	7	11.9	0.962	15	12.9	0	0.0	0.358	1	3.0	0.113	1	3.3	0.190
*cat-TC*	16	12.5	9	13.0	7	11.9	0.841	16	13.8	0	0.0	0.360	**0**	**0.0**	**0.011**	**0**	**0.0**	**0.023**
ERY	124	96.9	67	97.1	57	96.6	1.000	112	96.6	12	100	1.000	32	97.0	1.000	30	100.0	1.000
*erm (B)*	126	98.4	69	100	57	96.6	0.211	115	99.1	11	91.7	0.179	32	97	0.451	30	100.0	1.000
*mef (A)*	53	41.4	27	39.1	26	44.1	0.572	45	38.8	8	66.7	0.062	**31**	**93.9**	**0.000**	**30**	**100.0**	**0.000**
*msr (D)*	53	41.4	27	39.1	26	44.1	0.572	45	38.8	8	66.7	0.062	**31**	**93.9**	**0.000**	**30**	**100.0**	**0.000**
Total ^d^	128		69		59			116		12			33			30		

^a^The classification of diseases combines clinical manifestations and pathogenic diagnosis and ultimately depends on the source of the strain. All patients identified as IPD had S. pneumoniae isolated from their normally sterile sites (cerebrospinal fluid/blood/pleural fluid/ascites). ^b^Number of resistant isolates in each antibiotic category. ^c^Percentage of resistant isolates in each antibiotic category. ^d^Number of total isolates in each group. ^e^Comparison of this serotype/ST with all other isolates. **Bold** indicates that the difference is statistically significant. PSSP, penicillin-susceptible S. pneumoniae; PISP, penicillin-intermediate S. pneumoniae; PRSP, penicillin-resistant S. pneumoniae; PNSP, penicillin-non-susceptible S. pneumoniae; AMX, amoxicillin; CRO, ceftriaxone; CTX, cefotaxime; MEM, meropenem; LVX, levofloxacin; MXF, moxifloxacin; SXT, trimethoprim-sulfamethoxazole; CLI, clindamycin; VAN, vancomycin; TET, tetracycline; CHL, chloramphenicol; ERY, erythromycin.

The antibiotic resistance phenotype varied by serotypes and STs. For example, serotype 19F had higher resistance to PEN (PNSP: 18.2%, n=6, p=0.000), CRO (33.3%, n=11, p=0.000), CTX (36.4%, n=12, p=0.000) and LVX (9.1%, n=3, p=0.022), and serotype 6B had higher resistance to CHL (50.0%, n=8, p=0.000). ST271 had higher resistance to CRO (33.3%, n=10, p=0.000), CTX (36.7%, n=11, p=0.000) and LVX (10.0%, n=3, p=0.026).

With regard to antibiotic resistance-related genes, almost all isolates carried the ERY resistance gene *erm(B)* (98.4%, n=126) and the TET resistance gene *tet(M)* (98.4%, n=126). Some isolates carried the CHL resistance gene *cat-TC* (12.5%, n=16) and the ERY resistance genes *mef(A)* (41.4%, n=53) and *msr(D)* (41.4%, n=53). In addition, *mef(A)* and *msr(D)* linkages were observed in this study (**Figure 3D**).

Amino acid substitutions in conserved catalytic motifs (SXXK) of the penicillin-binding proteins (PBPs) were observed in all β-lactam non-sensitive strains, as well as some frequent substitutions (PBP1a: T371, E397, N405, P432, N546, A550, TSQF574-577, L583, A585; PBP2b: T446, E476, T489; PBP2x: R254, M256, T338, I371, G382, R384, T401, N444, S531, L546, L565, S576. **Supplementary Table 1**). These substitutions in full length PBPs occurred more frequently in β-lactam non-sensitive strains (PBP1a: 9.76% [n=69.12 ± 1.40] *vs.* 1.45% [n=10.31 ± 5.81], p=0.008; PBP2b: 5.73% [n=38.98 ± 2.36] *vs.* 1.17% [n=8.00 ± 3.95], p=0.001; PBP2x: 10.54% [n=75.15 ± 1.61] *vs.* 3.13% [n=23.46 ± 9.52], p=0.018. **Supplementary Table 1**). After screening the substitution sites through the backward method-likelihood ratio test, the three sites of PBP2bAla617, PBP1aT540 and PBP1aT371 were introduced to obtain a predicted MIC value <0.06 μg/mL, with a consistency rate of 96.3% (sensitivity: 100%; specificity: 84.6%).

### Antibiotic Resistance Phenotypes

**Figure 5:**
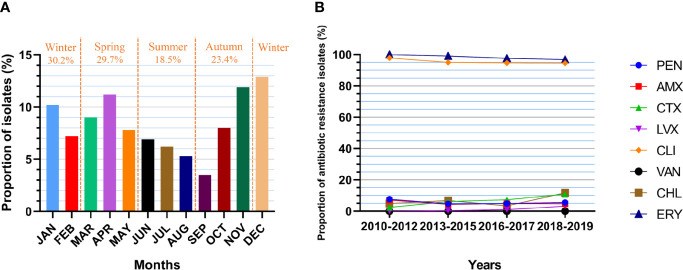
Isolate collection and antibiotic resistance of *S. pneumoniae*. **(A)** Proportion of cases from which *S. pneumoniae* was isolated in each month (n=993). JAN: 10.2%, FEB: 7.2%, MAR: 9.0%, APR: 11.2%, MAY: 7.8%, JUN: 6.9%, JUL: 6.2%, AUG: 5.3%, SEP: 3.5%, OCT: 8.0%, NOV: 11.9%, DEC: 12.9%. **(B)** Changes in the resistance rate of *S. pneumoniae* (numbers of isolates: n1 = 329, n2 = 458, n3 = 206, n4 = 128). PEN: 7.6%-4.6%-4.9%-5.5%, p=0.168. AMX: 7.0%-4.4%-4.9%-4.7%, p=0.255. CTX: 2.4%-6.1%-7.3%-10.9%, p=0.020. LVX: 0.0%-0.4%-1.0%-3.1%, p=0.223. CLI: 97.9%-95.0%-94.7%-94.5%, p=0.082. VAN: 0.0%-0.0%-0.0%-0.0%. CHL: 4.9%-6.8%-3.4%-11.7%, p=0.178. ERY: 100.0%-99.1%-97.6%-96.9%, p=0.016.

A total of 993 *S. pneumoniae* isolates were included in this analysis (**Table 5**). Based on the months of *S. pneumoniae* strains isolated, the proportion of patients with pneumococcal diseases in the whole year increased in autumn (23.4%, including 3.5% in SEP, 8.0% in OCT and 11.9% in NOV), with the largest proportion observed in winter (30.2%), followed by spring (27.9%) (**Figure 5A**). The prevalence of PNSP was 13.6%, including PISP (8.0%) and PRSP (5.6%), and most strains showed high resistance to ERY (99.1%) and CLI (95.9%). Few strains were resistant to CHL (5.4%), CTX (5.1%), AMX (5.3%) and LVX (0.4%), and no drug-resistant strains with resistance to VAN were observed. Compared to non-IPD strains, IPD strains had a higher rate of resistance to PEN (22.4% *vs.* 4.4%, p=0.000), AMX (14.9% *vs.* 4.6%, p=0.000) and CTX (11.9% *vs.* 4.6%, p=0.003) but a lower rate of resistance to CLI (88.1% *vs.* 96.4%, p=0.001) and CHL (3.0% *vs.* 5.6%, p=0.000). In addition, the rates of resistance to AMX and CTX in PNSP were significantly higher than those in PSSP (32.6% *vs.* 0.8%, p=0.000; 27.4% *vs.* 1.6%, p=0.000). The PEN resistance rate of the strains isolated from meningitis patients was significantly higher than that of strains isolated from non-meningitis patients (65.0% *vs.* 4.4%, p=0.000).

**Table 5:** Antibiotic resistance of *S. pneumoniae* isolates from 2010 to 2017 (retrospective study part, n=993).

Resistance			Diseases^a^					Penicillin resistance				
Phenotypes	Total^b^	%^c^	Non-IPD	%	IPD	%	P value	PSSP	%	PNSP	%	P value
PSSP	858	86.4	**809**	**87.4**	**49**	**73.1**	**0.001**					
PISP	79	8.0	76	8.2	3	4.5	0.276					
PRSP	56	5.6	**41**	**4.4**	**15**	**22.4**	**0.000**					
AMX	53	5.3	**43**	**4.6**	**10**	**14.9**	**0.000**	**7**	**0.8**	**44**	**32.6**	**0.000**
CTX	51	5.1	**43**	**4.6**	**8**	**11.9**	**0.003**	**14**	**1.6**	**37**	**27.4**	**0.000**
LVX	4	0.4	4	0.4	0	0.0	0.590	3	0.3	1	0.7	0.505
CLI	952	95.9	**893**	**96.4**	**59**	**88.1**	**0.001**	819	95.5	133	98.5	0.106
VAN	0	0.0	0	0.0	0	0.0		0	0.0	0	0.0	
CHL	54	5.4	**52**	**5.6**	**2**	**3.0**	**0.000**	51	5.9	3	2.2	0.076
ERY	984	99.1	918	99.1	66	98.5	0.427	849	99.0	135	100.0	0.378
Total^d^	993		926		67			858		135		

^a^The enrolment of invasive pneumococcal disease (IPD) patients was based on clinical diagnosis, and these patients had S. pneumoniae isolated from their normally sterile sites (CSF/blood/pleural fluid/ascites). ^b^Number of resistant isolates in each antibiotic category. ^c^Percentage of resistant isolates in each antibiotic category. d. Number of total isolates in each group. Bold indicates that the difference is statistically significant. PSSP, penicillin-susceptible S. pneumoniae; PISP, penicillin-intermediate S. pneumoniae; PRSP, penicillin-resistant S. pneumoniae; PNSP, penicillin-non-susceptible S. pneumoniae; AMX, amoxicillin; CTX, cefotaxime; LVX, levofloxacin; CLI, clindamycin; VAN, vancomycin; CHL, chloramphenicol; ERY, erythromycin.

Over the decade, the resistance of *S. pneumoniae* isolates to CTX increased significantly (p=0.020), with a resistance rate of 2.4% in 2010-2012, 6.1% in 2013-2015, 7.3% in 2016-2017 and 10.9% in 2018-2019 (**Figure 5B**). In addition, the resistance to ERY declined significantly (p=0.016), with a resistance rate of 100.0% in 2010-2012, 99.1% in 2013-2015, 97.6% in 2016-2017 and 96.9% in 2018-2019. The resistance of the isolates to other antibiotics did not change significantly over time.

## Discussion

In this study, we describe the application of whole-genome NGS approach for comprehensive identification of the molecular characteristics including serotype, ST, conservation of virulence factor gene and the presence of antibiotic resistance gene of pneumococcal isolates circulating in Southwest China, during 2017-2019. We demonstrate that pneumococcal serotypes 19F, 19A, 6B, 6A, and 14 are the top 5 serotypes in the region, show that most protein candidate genes are highly conserved in isolates, about 10% of the isolates are PNSP phenotype, about 90% of the isolates are multidrug resistant (MDR) strains, and *S. pneumoniae* resistance to CTX have increased significantly over the past decade.

The most prevalent serotypes (19F, 19A, 6B, 6A, and 14) in our regions were similar to those in other regions of China, such as Chongqing (19F, 6A/B, 19A) (Yu et al., 2019), Eastern region of Shanghai (19F, 19A, 6A, 14, 6B) (Wang et al., 2020b), Northern region of Beijing (19F, 19A, 23F, 14, 6A) (Wang et al., 2020a) and Southern region of Liuzhou (19F, 6B, 19A, 23F, 14) (Li et al., 2018), suggesting a general distribution of pneumococcal serotypes in China. However, the prevalence of pneumococcal serotypes in our region was slightly different from those in developing countries and regions e.g. Thailand (6B, 23F, 14) (Hocknell et al., 2019), Malaysia (14, 6B, 19A, 6A) (Arushothy et al., 2019), Mexico (19A, 3, 15B, 19F) (Echaniz-Aviles et al., 2019) and northern Russia (19F, 23F, 6A) (Vorobieva S Jensen et al., 2020), and were totally different from those in developed countries e.g. the USA (35B, 3, 11A/D) (Suaya et al., 2020), the UK and Ireland (3, 8, 15A) (Pick et al., 2020), and Japan (12F, 3, 23A) (Yanagihara et al., 2021). The similarity or discrepancy in the distribution of serotypes may relate to the following factors: 1) native *S. pneumoniae* circulating in various regions has different evolutionary directions, resulting in different capsule genes; 2) population of different races has different susceptibilities to different serotypes of *S. pneumoniae*; 3) the PCV vaccination status of the populations in different regions varies, which may be the most important factor.

Serotype replacement has been observed in regions where PCVs are widely available (Richter et al., 2013; Nakano et al., 2016). Compared with our previous data (Yan et al., 2019), the PCVs-covered serotypes decreased slightly in this region (PCV7: 68.7% *vs.* 50.0%; PCV10: 69.7% *vs.* 50.8%; PCV13: 93.3% *vs.* 77.3%), which may attribute to the introduction of PCV13 in 2017. The serotype coverage of PCV13 here is slightly lower than that in other regions in China (Beijing: 83.2%; Shanghai: 91.8%) (Wang et al., 2020a; Wang et al., 2020b) but remains higher than that in other developed countries (Spain: 52%; USA: 41.4%; Japan: 37.5%) (Richter et al., 2013; Nakano et al., 2016; Ciruela et al., 2018). In agreement with the results reported by Wang et al. (Wang et al., 2020a), the present study also shows that the serotype coverage for PCVs was higher for IPD strains than non-IPD strains, and serotype 14 strain was several times more frequently isolated in IPD patients than non-IPD patients. These results suggest that the PCV13 vaccine remains an effective means to control pneumococcal infection in this region, especially for invasive infection.

The present study shows that ST271, ST320, ST90 and ST876 were the most prevalent STs in Southwest China, which were similar to those in other regions in China (Li et al., 2018; Wang et al., 2020b). The present study further revealed 8 novel STs which accounted for 6.25% of all isolates (8/128), suggesting a higher variation and mutation rate for *S. pneumoniae* over other respiratory pathogenic bacteria e.g. *S. aureus* and *H. influenzae*. Based on SLVs as a standard, the predominant prevalent PMEN clone was Taiwan^19F^-14, which constituted more than one-third of the total and was twice as prevalent in this region as in Shanghai (18.7%, 14/75) (Li et al., 2019). These results suggest that *S. pneumoniae* may have multiple origins in China. In addition, the prevalent STs in China were different from those in other countries, such as Japan (Nakano et al., 2016), northern Russia (Vorobieva S Jensen et al., 2020), Iran (Talebi et al., 2016) and Latin America (Moreno et al., 2020), strongly suggesting the diversity of *S. pneumoniae* on a global scale.

*S. pneumoniae* harbours dozens of virulence factors, including the capsular polysaccharide, and adhesion factors e.g. exoenzymes, IgA1 protease, metal ion uptake factors, protease, and toxin factors, which were implicated in the pathogenesis of pneumococcal disease. Colonization is thought to be the first step for host infection (Weiser et al., 2018), and some of the key factors include: 1) pilus-1 (RrgA) and PfbA, which are implicated in first contact with the epithelium and epithelial receptors; 2) Phts, Eno and PepO, which can interact with the complement system; 3) NanA, BgaA and StrH, which function in mucus degradation; 4) PiaA, PiuA and PsaA, which harbor metal binding activity; 5) CbpE, which is related with the impairment of neutrophil activity; and 6) pneumolysin (Ply), which can damage the epithelium cells and have pro-inflammatory effects. The present work demonstrate that all isolates carry the above key factor genes, namely, *phtE, eno, pepO, nanA, bgaA, strH, piaA, piuA, psaA, cbpE* (*pec*) and *ply*, and carried at least one *rrgA* and *pfbA*, suggesting their indispensability in the pathogenic process of the isolates. After colonization, a variety of other virulence factors are involved in the following processes of pathogenesis, including adherence-related proteins e.g. LytA/B/C, CbpD/G, Lmb, SrtA, SlrA, and NanB, immunomodulatory proteins e.g. Plr, CppA, IgA1 protease and ZmpB/C, invasiveness-related proteins e.g. PavA, Pilus-2, HysA, HtrA and Tig. In the samples of this study, the carriage of multiple virulence genes was associated with the serotypes and CCs of the isolates. It was found that the serotypes prevalent in this region exhibited high carriage rates for *Pilus-1* (19F/19A), *Pilus-2* (19F/19A), *nanB* (19F/19A/14), *iga* (19F), *cbpG* (6B) and *pfbA* (6B/6A/14), which may be the result of natural selection in regions with low vaccination rates. The CCs prevalent in this region showed high carriage rates for *lytB* (CC271/CC90/CC876), *Pilus-1* (CC271), *Pilus-2* (CC271), *nanB* (CC271/CC876), *iga* (CC271/CC90), *cbpG* (CC90) and *pfbA* (CC90/CC876), which may be the reason why these branches were common in clinical infections.

Some virulence factors of *S. pneumoniae* are also potential targets for recombinant protein vaccines. Compared with PCVs, recombinant protein vaccines have lower cost and stronger immunogenicity and are not restricted by serotypes (Masomian et al., 2020). According to the target gene sequences of recombinant protein vaccines reported in the literature, we examined 27 protein vaccine candidate genes and found that more than 98% of the strains carried these genes, and 16 genes had sequence identity more than 98%. These results suggest that these protein antigens are widely conserved in pneumococcal isolates in Southwest China and may have high application potential in the development of recombinant protein vaccines.

PEN has historically been the first-line choice of antibiotics to treat *S. pneumoniae* infections. The present work demonstrates that the resistance rate of the isolates to PEN was less than 5% in non-meningitis patients, coincident with the results reported in other regions in East Asia (China: 0.7%-2.2%, Korea: 1.0%, Japan: 1.1%-3.8%) (Toda et al., 2018; Wang et al., 2019; Kim et al., 2020). However, 50%-70% of the meningitis strains were resistant to PEN using the MIC breakpoint ≥ 8 μg/mL according to CLSI 2020 criteria. These results suggest that PEN can continue to be used as a therapeutic drug for non-meningitis *S. pneumoniae* at higher concentrations, but it is not suitable for meningitis strains.

Altered PBP1a, PBP2b and PBP2x are the most important PBPs for β-lactam resistance among clinical isolates (Hakenbeck et al., 2012; Zhou et al., 2016). In this study, amino acid substitutions in conserved catalytic motifs STMK of PBPs were found in all β-lactam non-sensitive strains. The consistency, sensitivity and specificity of using the PBP2bAla617, PBP1aT540 and PBP1aT371 substitutions to predict MIC values less than 0.06 μg/mL were 96.3%, 100% and 84.6%, respectively. These results suggest that detection of the mutations may be helpful to predict the PEN resistance phenotype of isolates. It was found that IPD strains were more likely to have AMX and cephalosporins resistant phenotype than non-IPD strains, and the resistant rate of IPD strains to commonly used cephalosporins has also increased over the decade. These results suggest that MEM in combination with VAN can be chosen for the treatment of IPD before antibiotic susceptibility results are issued.

Macrolides were once heavily used in China because they were empirically used to cover *Mycoplasma pneumoniae* in children with infectious diseases and because of their safety in children and the exemption of a skin test before medication. According to the data from the China Antimicrobial Resistance Surveillance System in 2019, the resistance rate of *S. pneumoniae* to ERY was very high, with an average of 95.6% across the country. Macrolide resistance in *S. pneumoniae* is mainly mediated by: 1) target modification by a ribosomal methylase encoded by the *erm*(*B*) gene; and 2) a membrane efflux pump encoded by the *mef*(*A/E*) gene. The *erm*(*B*) gene was commonly detected in populations in mainland China, Taiwan, Japan, Thailand, Sri Lanka and South Korea, while *mef*(*A*) was more commonly detected in populations in Hong Kong, Singapore, Philippines, India and Malaysia (Kim et al., 2012). In this study, *erm*(*B*) and *mef*(*A*) were detected in 98.4% and 41.4% of the strains, respectively, which may be responsible for the heavy ERY resistance burden in this region. Although 14 ministries of China jointly issued a 5-year national action plan to control antimicrobial resistance (NAP) in 2016, 51.4% of prescriptions related to antibiotics, including the use of macrolides, remain inappropriate (Zhao et al., 2021). Similar to the results from other reports (Li et al., 2019; Wang et al., 2020b), the isolates were highly resistant to CLI in this work, thus, CLI may be not suitable for the treatment of *S. pneumoniae* infections. In addition, due to the potential toxic effects on children, the use of CHL (bone marrow suppression), TET (enamel hypoplasia), quinolones (achondroplasia) and sulphonamides (jaundice) is prohibited or restricted at different ages. These data suggest that macrolides are no longer suitable for the treatment of *S. pneumoniae* infections in China.

In this study, correlations between STs (CCs) and serotypes, virulence genes and antimicrobial resistance-related genes were observed, and some STs showed obvious aggregation in the branches of the RAxML tree. ST271, ST320, ST90 and ST876 were specifically associated with serotypes 19F, 19A, 6B and 14, respectively, which was quite similar to the results in a previous report (Li et al., 2018). Serotype 19F/ST271, as the predominant prevalent strain in the region, showed higher PEN non-sensitivity and was more resistant to CTX, CRO, and SXT, which is consistent with Margaret’s report (Ip et al., 2015). Moreover, serotype 19F/ST271 was more likely to carry the virulence factors encoding genes *pilus-1*, *pilus-2*, *nanB* and *iga* as well as the macrolide resistance genes *mef*(*A*) and *msr*(*D*). These results suggest that some strains or clones may have evolved many important features including resistance to antibiotics and pathogenesis-related strategies to have a competitive advantage in adapting to the host. Strategies e.g. vaccination are therefore needed to control the widespread of these prevalent strains or clones.

The main strengths of the present study are the multicentre design and the utility of whole-genome NGS technology to characterize the prevalence of pneumococcal strains and analysed the encoding genes associated with pneumococcal virulence factors, potential protein vaccine candidates, and the antibiotic resistance proteins.

This study has some limitations. First, the sample size is relatively small which may undermine the representativeness of the prevalent strains in Southwest China. Second, since the strains isolated during 2010-2017 were unavailable, the evolution of genes associated with drug resistance cannot be compared and analysed. Third, only a very limited data mining was currently performed, further studies regarding the correlations between the genome characteristics and strain phenotypes or clinical indicators are warranted.

In conclusion, serotypes 19F, 19A, 6B, 6A, and 14 were most prevalent strains responsible for childhood pneumococcal disease in Southwest China. Some strains or clones, e.g. Taiwan^19F^-14 have evolved many antibiotic resistance strategies against PEN, CTX, CRO, and SXT. Given the high coverage rate of PCV13 and the worrisome non-susceptibility rate of pneumococcal isolates to antibiotics, vaccination with PCV13 may be beneficial in this region. Since pneumococcal virulence protein encoding genes are highly conserved in pneumococcal isolates, the development of virulence protein-based vaccines is also expected.

## Data Availability Statement

The datasets presented in this study can be found in online repositories. The names of the repository/repositories and accession number(s) can be found below: https://www.ncbi.nlm.nih.gov/genbank/, PRJNA681770 https://www.ncbi.nlm.nih.gov/genbank/, PRJNA656156 https://www.ncbi.nlm.nih.gov/genbank/, PRJNA643306.

## Ethics Statement

The studies involving human participants were reviewed and approved by Clinical Trial Ethics Committee of West China Second University Hospital, Sichuan University. Written informed consent to participate in this study was provided by the participants’ legal guardian/next of kin.

## Author Contributions

Conceptualization: ZY and YC. Methodology: ZY. Software: ZY and SL. Validation: YC. Formal analysis: ZY, XH, WT, SL, WC and MS. Investigation: ZY, XH, WT and MS. Resources: WZ. Data Curation: ZY and WC. Writing - Original Draft: ZY. Writing - Review and Editing: YC and KW. Visualization: ZY. Supervision: YJ. Project Administration: WZ. Funding Acquisition: YC, KW and YJ. All authors contributed to the article and approved the submitted version.

## Funding

This work was supported by the Science & Technology Department of Sichuan Province (No. 2020YFS0100, No. 2020YFS0103), the Health Commission of Sichuan Province (No. 17ZD005, No. 19PJ227), the Chengdu Science & Technology Bureau (No. 2019-YF05-01178-SN), the Fundamental Research Funds for the Central Universities (No. SCU2019C4198), the Science & Technology Department West China Second University Hospital, Sichuan University (No. KL044, No. KZ062), Cadres Healthcare Research Projects in Sichuan Province (No. 2021-1703), Innovation Group Project provided by Education Department of Guizhou Province (QiankeheKYzi [2020]019), and Science and Technology Project of Guizhou Province (Qiankehezhicheng [2020]4Y201). The funders had no role in study design, data collection and analysis and interpretation of the data.

## Conflict of Interest

The authors declare that the research was conducted in the absence of any commercial or financial relationships that could be construed as a potential conflict of interest.

## Publisher’s Note

All claims expressed in this article are solely those of the authors and do not necessarily represent those of their affiliated organizations, or those of the publisher, the editors and the reviewers. Any product that may be evaluated in this article, or claim that may be made by its manufacturer, is not guaranteed or endorsed by the publisher.

## Supplementary Material

Supplementary Table 1 Association of β-lactams resistant, serotypes, STs and amino acid substitutions in PBPs. The sequences were aligned according to the corresponding amino acid sequences of R6. Catalytic motifs in PBP1a: Ser370-Thr371-Met372-Lys373 (STMK). Frequent substitutions in PBP1a conferring high MIC values: Glu397, Asn405, Pro432, Thr540, Asn546, Ala550, Thr574-Ser575-Gln576-Phe577 (TSQF), Leu583 and Ala585. Catalytic motifs in PBP2b: Ser386-Val387-Val388-Lys389 (SVVK). Frequent substitutions in PBP2b conferring high MIC values: Gln427, Thr446, Glu476, Thr489 and Ala619. Catalytic motifs in PBP2x: Ser370-Thr371-Met372-Lys373 (STMK). Frequent substitutions in PBP2x conferring high MIC values: Arg254, Met256, Ile371, Gly382, Arg384, Thr401, Asn444, Ser531, Leu546, Leu565 and Ser576. No amino acid substitution in SXN or KXG catalytic motifs was observed in this study. a. According to the Performance Standards for Antimicrobial Susceptibility Testing M100, 2020. The phenotype of penicillin resistance varies depending on the source of the strain. b. The concentration unit is μg/mL. c. Gaps were observed in this alignment. CSF, cerebrospinal fluid; BALP, bronchoalveolar lavage fluid; PEN, penicillin; CRO, ceftriaxone; CTX, cefotaxime; CXM, cefuroxime; FEP cefepime.
